# The Role of the Gut Microbiome in Type 2 Diabetes Mellitus

**DOI:** 10.3390/ijms262311412

**Published:** 2025-11-26

**Authors:** Rahaf Mashal, Amnah Al-Muhanna, Salma Khader, Aiman Khudair, Ahmed Khudair, Alexandra E. Butler

**Affiliations:** 1 School of Medicine, Royal College of Surgeons in Ireland—Medical University of Bahrain, Busaiteen P.O. Box 15503, Bahrain; 20204439@rcsi-mub.com (R.M.); 20205504@rcsi.com (A.A.-M.); 21200514@rcsi.com (S.K.); 20204862@rcsi.com (A.K.); 20204861@rcsi.com (A.K.); 2 Research Department, Royal College of Surgeons in Ireland—Medical University of Bahrain, Busaiteen P.O. Box 15503, Bahrain

**Keywords:** diabetes, gut microbiome, dysbiosis

## Abstract

The gastrointestinal tract in humans hosts trillions of microorganisms, collectively termed the gut microbiota, which perform essential physiological processes and roles, including nutrient metabolism and immunomodulation. Influenced by genetics, age, diet, medication, and the environment, the disruption of this system leads to dysbiosis, which has been linked to a range of diseases, notably type 2 diabetes mellitus (T2DM). As the global prevalence of T2DM continues to trend upwards, research investigating and highlighting the influence the gut microbiome exerts on this disease is warranted. The literature was examined regarding microbial metabolites and metabolic signaling pathways, as well as interventions relating to diet, prebiotics, probiotics, pharmacological agents, and fecal microbiota transplantation (FMT). The gut microbiome, through its effects on insulin resistance, inflammation, bile acid signaling, and glucose–lipid metabolism, impacts the development and progression of T2DM. Furthermore, patients with T2DM have demonstrated reduced microbial diversity, depletion of butyrate-producing bacteria, and an increase in pathogenic species. Interventions including high-fiber diets, metformin, probiotics, and FMT were shown to enrich beneficial microbes and improve metabolic outcomes. Targeted modulation of the microbiome, such as through next-generation probiotics and CRISPR-based therapies, may enhance metabolic control in the context of the future of personalized medicine. This review investigates the intricate relationship between the gut microbiome and T2DM, emphasizing its role in disease pathogenesis, the factors that may impact the microbiome in these patients, as well as therapeutic approaches toward its management.

## 1. Introduction

**Figure 1:**
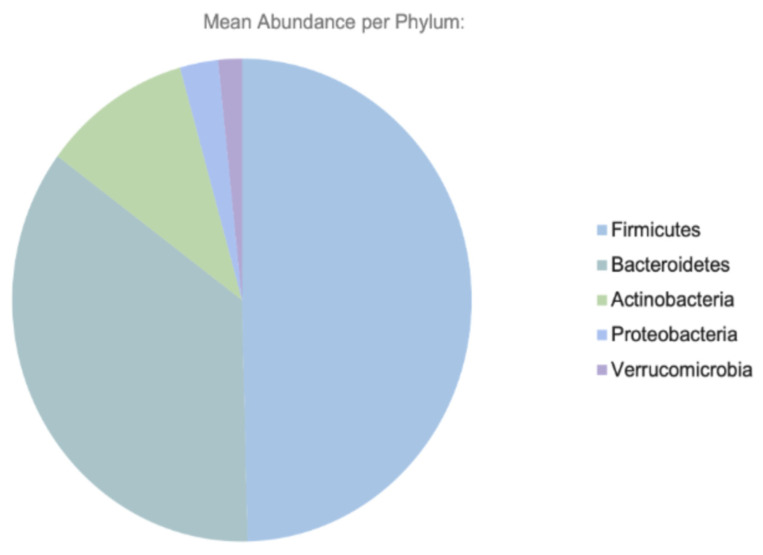
Major phyla of the human GI tract.

The gastrointestinal (GI) tract in humans is home to many microorganisms, collectively termed the gut microbiota [1]. This diverse community comprises organisms from the domains Bacteria, Archaea, and Eukarya [2,3,4]. These microorganisms, which accompany us from birth and throughout life, colonize the GI tract and perform a wide range of essential functions [5]. The term gut microbiome refers not only to the microorganisms (microbiota) residing in the gastrointestinal tract but also to their structural environment, genetic material, and metabolic products [6,7,8]. These microorganisms, estimated to exceed 10^14^ in number, are most densely concentrated in the colon [2,9]. Of the approximately 1150 microbial species capable of colonizing our GI tract, each individual has 160 species at a minimum, with the 5 major phyla being *Bacteroidetes*, *Actinobacteria*, *Firmicutes*, *Proteobacteria*, and *Verrucomicrobe* [10]. These microorganisms are categorized into several major phyla, predominantly Bacteroidetes, Firmicutes, Actinobacteria, Proteobacteria, and Verrucomicrobia (Figure 1). Although microbiota are also found on the skin, in the oral cavity, and within the lungs, the gut microbiota is considered the most crucial influence upon maintenance of overall health [7,11,12].

**Figure 2:**
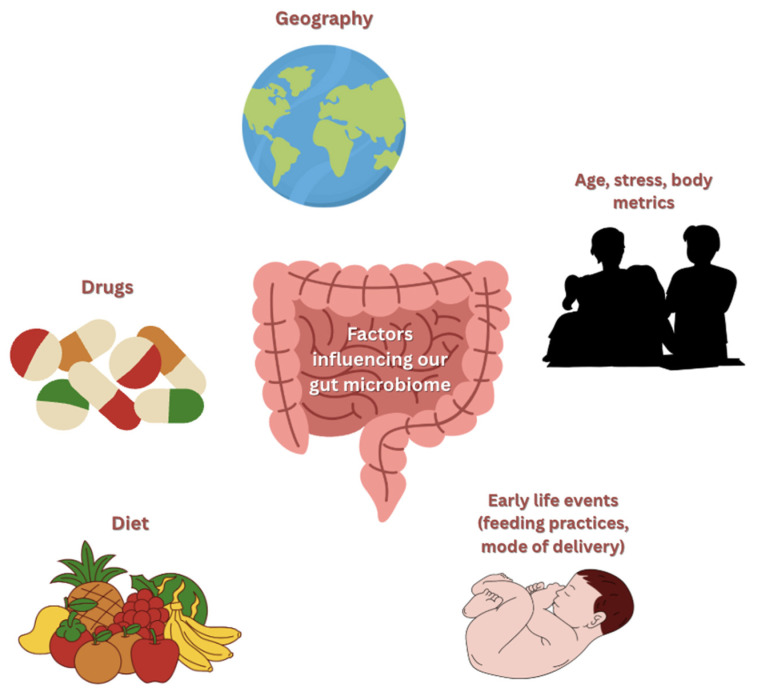
Factors influencing the gut microbiome.

The gut microbiome plays a central role in numerous physiological processes, including nutrient metabolism, immunomodulation, drug metabolism, maintenance of the integrity of the GI mucosal barrier, and defense against pathogens [10,13]. While the human genome contains approximately 23,000 genes, the gut microbiome’s genetic repertoire is immense, encoding over 3 million genes [14]. While a portion is inherited, as shown in twin studies, many factors heavily influence its composition, such as the drugs ingested, age, stress, diet, body metrics, geography, and early life events, such as feeding practices and mode of delivery in infancy, as illustrated in Figure 2 [14,15,16].

Just as physiological processes in the body can become dysregulated, the microbiome itself may become subject to such an imbalance in its function or overall composition, a state referred to as dysbiosis [6,17]. Dysbiosis can present in several forms, including a reduction in beneficial bacteria, an overgrowth of pathogenic bacteria, and a reduction in overall bacterial diversity [18]. Studies have demonstrated an association between dysbiosis and various diseases, including diabetes, colorectal cancer, and cardiovascular disease [6,19,20,21,22,23].

Among the many diseases influenced by the gut microbiome, type 2 diabetes mellitus (T2DM), as a growing global health epidemic, is a critical one. Globally, it is estimated that T2DM affects 589–828 million people, causing 4.2 million deaths in 2019, according to the International Diabetes Federation (IDF) [24,25]. Furthermore, a projected rise in the age-standardized global prevalence of T2DM from 5.9% in 2021 to 9.5% in 2050 is predicted, an alarming increase of 61.2% [26].

Encompassing 90–95% of all global diabetes cases, T2DM is characterized by the impaired secretion of insulin from beta cells in the pancreas, along with reduced insulin sensitivity in peripheral tissues, such as skeletal muscle, liver, and adipose tissue [24,25]. Multiple factors influence the risk of an individual developing T2DM; there are non-modifiable risk factors, including family history and ethnicity, and modifiable risk factors, such as a sedentary lifestyle, obesity, and an unhealthy diet [24,27,28]. Notably, the modifiable risk factors are contributors to the rise in T2DM in young adults, adolescents, and children [29]. Interestingly, the role of the gut microbiome in terms of its relation to T2DM has only been recently investigated, where it has been found to impact the outcome, progression, and even the causation of diabetes [30,31,32]. These advancements in the field are aided by advancements in high-throughput sequencing and bioinformatics, which enable in-depth study of the role of the microbiome and its overall impact on various bodily processes [33].

T2DM and its association with the gut microbiota have been reported in a number of studies [22,34,35,36]. Of note, T2DM patients were shown to have moderate dysregulation of their gut microbiota, including a reduction in butyrate-producing bacteria with an increase in opportunistic pathogens [22]. Furthermore, certain species of bacteria, such as *Lactobacillus* and *Clostridium* species, were associated with alterations in fasting glucose, glycosylated hemoglobin, and plasma triglycerides [34,37]. Notably, confounding factors, such as diet, health status of the individual, and medication usage, make it challenging to identify a consistent microbiota composition that is common across T2DM patients [9,38]. Moreover, a clear cause-and-effect relationship cannot be established due to the variation in study design and observational methodologies [39]. Nevertheless, the gut microbiome’s unique fingerprint-like composition may serve as a valuable tool for identifying those at risk and as a prognostic indicator to guide personalized therapeutic interventions [40].

Given the rising burden of T2DM and the increasing recognition of the gut microbiome as a key player, a review of the current literature is critical and clinically relevant. This review aims to synthesize the latest findings to deepen the understanding of the link between the gut microbiome and T2DM while highlighting recent advancements and exploring future clinical implications.

## 2. Gut Microbiome: Composition and Function

The microbiome in the gut essentially functions as an organ in its own right [41,42]. This vast community of microbiota performs a range of digestive and metabolic functions to influence host health. Among these functions are the digestion of dietary substrates, production of signaling molecules for neuro-immunoendocrine activity (as in the brain–gut axis), and anti-inflammatory processes involved in immune regulation [43].

The roles of the microbiome within the gut can be subdivided into metabolic, structural, and protective. Within the domain of metabolism, and carbohydrate metabolism in particular, the microbiota source nutrition for both the host and themselves. Carbohydrates that escape digestion are used as substrates in microbial fermentation, resulting in the synthesis of short-chain fatty acids (SCFAs) such as butyrate and acetate [13]. Carbohydrates directly provide nutrition for microbiota, and the SCFAs derived from carbohydrate metabolism serve as energy sources for the host.

Another key function is vitamin production, either via vitamin-K-producing bacteria, such as *Bacteroides fragilis* and *Enterobacter agglomerans*, or vitamin B5 and B12 synthesis [44]. Several gut-resident bacterial species are capable of de novo vitamin B12 synthesis, including *Propionibacterium freudenreichii*, *Clostridium* spp., *Bacteroides fragilis*, and certain strains of *Lactobacillus reuteri*. Similarly, vitamin B5, a precursor to coenzyme A, is produced by various commensal bacteria, such as *Bifidobacterium adolescentis*, *Bacteroides thetaiotaomicron*, *Lactobacillus plantarum*, and *Enterococcus faecium.* Although the consequences of vitamin deficiency in disease are well documented, further insight is required into the consequences of fluctuations in vitamin-producing microbiota in the gut.

Protective mechanisms encompass immunomodulation and defense against pathogens. Immunomodulation may be observed through the action of fermentative bacteria; they produce histamine decarboxylase, which converts L-histidine to histamine. This bacterial histamine then acts as an immunoregulatory signal, suppressing tumor necrosis factor (TNF) production and thus alleviating inflammatory effects [45]. To aid in pathogen defense, a certain physiological state must be maintained, termed colonization resistance [44]. The gut microbiota serves as a protective barrier by competing with invading pathogens for nutrients and epithelial binding sites, thereby limiting their colonization and maintaining microbial community stability.

The architecture of the gut supports microbiome residence; mucosal integrity is maintained by bacterial suppression of inflammation—for example, via regulatory T cells or butyrate. Indole, a key metabolite, has been found to induce genes responsible for tight junction organization, adherens junctions, and mucin production, thus fortifying the mucosal barrier [46].

Diversity in the gut is attributed to the trillions of organisms that reside there. *Firmicutes* dominate, followed by *Bacteroidetes* and *Actinobacteria* with a smaller proportion of *Fusobacteria* and *Proteobacteria* [43]. Beyond bacteria, various fungi, archaea, and protists are also present [47]. However, these populations do not remain static; as a neonate grows, from childhood to adolescence and onwards to adulthood, so does the microbiome, with increasing diversity [11]. As the immune system develops, the microbiome plays a crucial role in stability and resilience. For example, antibiotic use in infancy has been suggested to influence immunological deficits later in life, such as atopic diseases [48]. In accordance with the hygiene hypothesis, preserving diversity is a necessity [49].

Diversity highlights distinct characteristics within communities: richness, which is the number of distinct taxa, and evenness, the distribution of individual organisms among those taxa. In essence, it reflects not only how many organisms exist but also the harmony of their coexistence. Further classification yields alpha and beta diversity: alpha diversity measures the compositional diversity within one sample—for example, an individual’s gut microbiome—and beta diversity compares the contrasting microbe abundances between samples—two microbiomes shaped by different dietary or geographical influences, with distinct microbial compositions [11,50].

As an example of geographical influences, non-Western populations tend to show a higher mean relative abundance of *Prevotella* species compared to a higher abundance of *Clostridium* and *Bacteroides* in the West [51]. The dominant *Bacteroidetes* generally increase with age, but they are observed to decrease in healthy older Indonesians and Indians [11]. To map these regional discrepancies and dissect their functional and taxonomic profiles, methods such as 16S rRNA sequencing have long been utilized; however, as knowledge grows, new tools are required to bridge the gaps, and this is reflected by breakthroughs in techniques and methods, such as genome-resolved metagenomics [52].

Global variation in the microbiota can be measured, and differing profiles can be accounted for based on factors such as diet, sanitation, and environment. Significant deviations from established norms can provoke disruptions in function and precipitate disease. Understanding how and why deviations cause disease is critical to prevention.

Findings have long suggested an enduring influence of dysbiosis—defined as a disruption in the composition and function of the microbiome—during early development, as evidenced by the well-documented association between antibiotic exposure in infancy and the later onset of atopic conditions. Dysbiosis has also been implicated in a wide range of long-term outcomes, including altered trajectories of disease progression and compromised health [53].

Dysbiosis can be distinguished as either a loss of diversity, due to the eradication of beneficial microbiota, or an increased abundance of pathogenic species [47]. The role of dysbiosis in disease, typified by inflammatory bowel disease, is not confined to the gut, with metabolic disorders (obesity, type 2 diabetes) and allergies as additional consequences [54]. Neurological manifestations may also occur, with ongoing research into conditions such as autism and Alzheimer’s [55].

Diet is implicated in the geographical variation in microbiota and is thus linked to disease susceptibility [56]. Host immunity and barrier function are also affected, influenced by the use of antibiotics and non-steroidal anti-inflammatory drugs (NSAIDs), as well as the bacterial metabolism of various drugs. NSAID use coupled with proton-pump inhibitors provokes a significant change in the microbiome. Antibiotics are well known to be detrimental to the gut flora and contribute to rising resistance [57,58]. Emerging research indicates important associations with autoimmune and metabolic disorders. Therefore, rather than simply being viewed as a measure of health, the microbiome may be harnessed to preserve health.

## 3. Discussion: Pathophysiological Link Between the Gut Microbiome and Type 2 Diabetes 

### 3.1. Mechanisms of Microbiome-Induced Insulin Resistance

Insulin resistance in type 2 diabetes arises in part from gut microbial dysbiosis. Patients with early insulin resistance tend to show reduced richness and diversity of butyrate-producing bacteria—including *Christensenellaceae*, *Marvinbryantia*, and various *Ruminococcaceae*—which correlates with increased Homeostasis Model Assessment for Insulin Resistance (HOMA-IR) values across cohort studies [59]. Lower levels of *Faecalibacterium prausnitzii* (a genus of bacteria) and other SCFA producers compromise intestinal barrier function, permitting bacterial lipopolysaccharide (LPS) translocation into the circulation. Once systemic, LPS activates Toll-like receptor 4 (TLR4) pathways, resulting in chronic low-grade inflammation and serine phosphorylation of Insulin Receptor Substrate (IRS), thus impairing insulin signaling in liver, muscle, and adipose tissues [34].

Mechanistic reviews further underscore the role of SCFAs in maintaining gut integrity; depletion of SCFAs, like butyrate, reduces regulatory T cell activity and tight junction protein expression, such as zonula occludens-1 (ZO-1), worsening permeability [31]. In support of this mechanism, butyrate deprivation in murine epithelial models resulted in a 50% reduction in ZO-1 expression and increased paracellular permeability, demonstrating a causal link between butyrate levels and barrier integrity [60]. Meanwhile, the microbiome acts like an endocrine organ, modulating host metabolic pathways through the production of metabolites that influence distal organs through receptor pathways and neural communication [61]. Notably, metformin’s ability to enrich *Akkermansia muciniphila* (a mucin-degrading bacterium of the intestinal niche, exerting beneficial effects on the host metabolic profile) and SCFA producers appears to have insulin-sensitizing effects in both humans and animal models [62]. Taken together, these data portray a dynamic system in which dysbiosis, barrier leakage, inflammation, and disrupted SCFA signaling converge to initiate and maintain insulin resistance in T2DM.

### 3.2. Influence on Glucose and Lipid Metabolism

Gut microbial fermentation of dietary fiber produces short-chain fatty acids (SCFAs), such as acetate, propionate, and butyrate, which act through G protein-coupled receptor 41 (GPR41), also known as free fatty acid receptor 3 (FFAR3), and G protein-coupled receptor 43 (GPR43), also known as free fatty acid receptor 2 (FFAR2), to regulate insulin sensitivity and metabolic homeostasis. Beyond receptor binding, propionate suppresses hepatic gluconeogenesis by inhibiting pyruvate carboxylase, while butyrate enhances mitochondrial function and fatty acid β-oxidation via AMPK and PGC-1α activation, resulting in improved insulin signaling [63]. These mechanisms collectively reduce hepatic glucose production and enhance peripheral glucose uptake [34]. Clinical studies confirm that diets high in fermentable fiber are linked to improved glycemic control and reductions in insulin resistance [64]. For example, a randomized controlled trial demonstrated that supplementation with inulin-type fructans improved insulin resistance and reduced fasting glucose levels in patients with T2DM [65].

Dysbiosis disrupts lipid metabolism by increasing energy harvest from the diet and promoting hepatic fat accumulation. This occurs through elevated de novo lipogenesis, impaired fatty acid oxidation, and altered cholesterol transport [66]. Acetate-producing Firmicutes promote hepatic triglyceride synthesis, while decreased abundance of butyrate-producing bacteria, such as *Faecalibacterium prausnitzii*, contributes to lipid accumulation and low-grade inflammation. Microbiota-targeted interventions, including prebiotics, probiotics, and fecal microbiota transplantation (FMT), have demonstrated metabolic benefits [61], with FMT from lean donors significantly improving peripheral insulin sensitivity in individuals with metabolic syndrome [67].

SCFAs also modulate appetite and glucose homeostasis by stimulating enteroendocrine L-cells to secrete anorexigenic hormones, such as GLP-1 and peptide YY (PYY), reinforcing insulinotropic effects, satiety, and delayed gastric emptying. This occurs through FFAR2-mediated intracellular calcium signaling and activation of proglucagon gene expression in L-cells [68]. Dysbiosis blunts SCFA-mediated incretin release, contributing to impaired satiety signaling, increased calorie intake, and progressive insulin resistance [62].

### 3.3. Effects on Bile Acid Metabolism and Incretin Hormones

The gut microbiota plays a central role in regulating bile acid (BA) composition and incretin hormone activity, two systems crucial for maintaining metabolic balance. Primary bile acids produced by the liver are transformed into secondary bile acids, such as deoxycholic acid (DCA) and lithocholic acid (LCA), by microbial enzymes, including bile salt hydrolases and 7α-dehydroxylases from Clostridium, Bacteroides, and Eubacterium species. These secondary bile acids act on nuclear receptors like farnesoid X receptor (FXR) and Takeda G-protein-coupled receptor 5 (TGR5), which are key regulators of glucose homeostasis, lipid metabolism, and energy expenditure [34]. Activation of intestinal FXR induces fibroblast growth factor 19 (F19), which suppresses hepatic gluconeogenesis, while TGR5 signaling on enteroendocrine cells enhances GLP-1 secretion, improving insulin sensitivity.

In T2DM, dysbiosis alters the bile acid pool, diminishing FXR and TGR5 signaling [9]. A shift toward primary conjugated bile acids reduces TGR5 activation, blunting GLP-1 secretion and worsening postprandial hyperglycemia. Additionally, a reduction in bile salt hydrolase-producing bacteria, such as *Lactobacillus* and *Bifidobacterium*, disrupts bile acid deconjugation and enterohepatic circulation, promoting metabolic inflammation and hepatic insulin resistance.

SCFAs also interact with bile acid pathways by modulating incretin hormone release via FFAR2, highlighting the interconnected nature of the gut–liver–hormone axis in glucose regulation [39,40].

### 3.4. Microbiome-Derived Metabolites: SCFAs, BCAAs, and Bile Acids

The gut microbiome produces a wide array of metabolites that regulate host metabolism, particularly in the context of T2DM. The most notable include SCFAs, branched-chain amino acids (BCAAs), and bile acids.

BCAAs—leucine, isoleucine, and valine—are consistently elevated in individuals with insulin resistance. Dysbiotic gut communities promote excessive BCAA biosynthesis or impair their degradation. A recent metagenomic analysis identified *Prevotella copri* as a major BCAA-producing species, showing that colonization with *P. copri* increases circulating BCAA levels and induces insulin resistance in humans [69]. Elevated circulating BCAAs activate mTOR and oxidative stress pathways in the liver and muscle, which interfere with insulin receptor signaling. Another microbiota-derived metabolite, trimethylamine-N-oxide (TMAO), has also been linked to metabolic dysfunction; it promotes insulin resistance by inducing hepatic inflammation and endothelial oxidative stress [70]. Studies show that modulating microbial BCAA production may improve insulin sensitivity and reduce metabolic dysfunction [34]. Bile acids, as discussed in Section 3.3, are transformed by microbial enzymes and interact with FXR and TGR5 receptors. Dysbiosis alters this balance, impairing bile acid-mediated hormonal and metabolic signaling pathways [71].

Furthermore, metabolomic profiling of patients with T2DM shows that dysregulated gut microbiota-derived metabolites are associated with systemic inflammation, mitochondrial dysfunction, and impaired intestinal barrier integrity, which may contribute to the progression of insulin resistance [72].

Fecal butyrate in particular is associated with reduced HbA1c and fasting glucose levels in individuals with T2DM [73,74], reflecting its potential therapeutic role in metabolic control.

Collectively, SCFAs, BCAAs, and bile acids form a triad of microbial metabolites that influence the pathophysiology of T2DM through hormonal, metabolic, and immune pathways. Targeting them may offer novel strategies for prevention and therapy. A summary of key gut microbiota-derived metabolites and their metabolic effects relevant to T2DM is presented in Table 1.

## 4. Factors Affecting the Microbiome in T2DM

### 4.1. Diet and Nutrition

Diet is one of the strongest modulators of gut microbiota composition and plays a major role in the development and progression of T2DM. Diets rich in fiber, polyphenols, and whole plant foods promote the growth of beneficial bacterial taxa, particularly short-chain fatty acid (SCFA)-producing species, such as *Faecalibacterium prausnitzii*, *Roseburia*, and *Bifidobacterium*. SCFAs act as key metabolic mediators that regulate host energy metabolism, enhance intestinal barrier integrity, and suppress systemic inflammation [75].

By contrast, Western dietary patterns—characterized by high intake of saturated fats and refined sugars, combined with low fiber consumption—are associated with gut dysbiosis, reduced microbial diversity, and an increased abundance of endotoxin-producing Gram-negative bacteria. This dysbiotic shift promotes low-grade systemic inflammation and impairs insulin signaling, both of which are key features of metabolic dysfunction in T2DM [76].

Interventional studies comparing isolated fiber supplements with whole-food dietary approaches have shown that plant-based diets result in greater microbial richness, higher SCFA production, and improved glycemic and lipid profiles. Fermented foods, such as yogurt, kimchi, and kefir, which provide live microorganisms and bioactive metabolites, further support microbial diversity and metabolic flexibility [77].

The mechanisms underlying these observations involve SCFA-mediated activation of free fatty acid receptors (FFAR2/3), leading to downstream activation of AMPK, enhanced peripheral glucose uptake, and GLP-1 secretion from L-cells. In summary, dietary quality plays a pivotal role in shaping the gut microbial ecosystem, which, in turn, regulates inflammation, gut integrity, and metabolic homeostasis. Nutritional strategies that emphasize microbiota-supportive foods may therefore represent a cornerstone in the prevention and management of T2DM.

### 4.2. Antibiotics and Medication

Pharmaceutical interventions, particularly antibiotics and glucose-lowering agents, exert profound and often underappreciated effects on gut microbiota composition and function. Antibiotics, especially broad-spectrum agents, are well documented to reduce microbial diversity, disrupt microbial balance, and increase the relative abundance of pathogenic or opportunistic taxa. Epidemiological data suggest a dose-dependent relationship between antibiotic exposure and the development of insulin resistance and T2DM, likely mediated by antibiotic-induced dysbiosis and gut barrier dysfunction.

Metformin, the first-line pharmacological treatment for T2DM, exhibits both therapeutic and microbiome-modulating effects. Beyond its classical mechanisms of action, involving hepatic gluconeogenesis inhibition and increased peripheral glucose uptake, metformin alters the intestinal microbiota, enriching beneficial taxa such as *Akkermansia muciniphila*, *Bifidobacterium*, and SCFA producers. These changes have been shown to contribute significantly to the drug’s glucose-lowering effects [78]. This relationship was demonstrated experimentally when fecal microbiota from metformin-treated donors were transplanted into germ-free mice, resulting in improved glucose tolerance compared with controls and confirming a causal contribution of metformin’s metabolic effects on microbiome remodeling [79].

However, not all medications exert beneficial effects on the gut microbiome. Proton pump inhibitors (PPIs), non-steroidal anti-inflammatory drugs (NSAIDs), statins, and selective serotonin reuptake inhibitors (SSRIs) have all been implicated in microbiota disruption. Long-term PPI use, for instance, has been associated with overgrowth of *Enterococcus* and *Clostridium difficile*, while NSAIDs may compromise mucosal integrity and increase intestinal permeability. These changes may indirectly promote metabolic inflammation and insulin resistance, thereby complicating glycemic control in patients with or at risk for T2DM.

Furthermore, recent studies have raised concerns about antibiotic resistance gene (ARG) enrichment within the gut microbiome following metformin use, suggesting that the drug’s interaction with the microbial ecosystem is not exclusively beneficial [80].

## 5. Dietary Interventions

### 5.1. Fibers

Diet plays a crucial role in shaping the gut microbiome and directly influences the development and management of T2DM. Fibers serve as essential substrates for the production of SCFAs. These are carbohydrate polymers and oligomers made up of monosaccharide units, which can vary in size and structure. Additional chemical groups, such as acetyl and methyl, may be included as well. Consequently, fibers manifest significant structural diversity and differ in physical characteristics, such as solubility and viscosity [81,82]. Fibers can be generally characterized as either dietary fibers or functional fibers. Dietary fibers are derived from grains, fruits, vegetables, and legumes [83]. Functional fibers consist of cellulose, some hemicellulose, and lignin, which primarily add bulk to aid intestinal transit [84,85].

### 5.2. Mediterranean Diets

The Mediterranean diet provides a balanced and sufficient intake of adequate nutrients, such as carbohydrates, proteins, fats, vitamins, and minerals, with emphasis on plant-based proteins, complex carbohydrates, and fiber, rich in monounsaturated and polyunsaturated fatty acids, while low in foods of animal origin. This diet supports the prevention and management of chronic non-communicable disease [86]. The Mediterranean diet encourages a high intake of bioactive food elements, which consist of monounsaturated fatty acids (MUFAs), polyunsaturated fatty acids (PUFAs), phytochemicals, dietary fibers, phytosterols, and probiotics. These bioactive elements are linked to numerous health benefits [87]. A large cohort study revealed that adherence to a Mediterranean diet is associated with an 11% reduction in the risk of developing T2DM [88,89].

## 6. Fecal Microbiome Transplantation (FMT)

Fecal microbiota transplantation (FMT) involves the transfer of a complete microbial community from a healthy donor to a recipient with gut dysbiosis, with the aim of restoring microbial balance and metabolic homeostasis. While FMT is an established therapy for recurrent *Clostridioides difficile* infection [90], its potential role in metabolic diseases, including type 2 diabetes mellitus (T2DM), has gained increasing interest due to its ability to favorably modify the gut microbiome [90,91,92]. Evidence suggests that FMT may improve glucose regulation and insulin sensitivity by increasing the abundance of beneficial taxa and enhancing short-chain fatty acid (SCFA) production. In a landmark randomized controlled trial, Vrieze et al. reported that individuals with metabolic syndrome who received allogeneic FMT from lean donors exhibited a significant improvement in peripheral insulin sensitivity six weeks after treatment, accompanied by an enrichment in butyrate-producing bacteria, such as *Roseburia intestinalis* [67]. Another study demonstrated reductions in HbA1c and favorable modulation of gut microbial composition following FMT in participants with metabolic dysfunction [9]. Interestingly, individuals with lower baseline microbial gene richness appeared to exhibit greater improvements, suggesting that gut microbial diversity may influence responsiveness to FMT [93].

Despite these promising findings, responses to FMT are highly variable and appear to diminish over time, indicating that sustained engraftment may require repeated administration or combination with adjunctive strategies. Challenges limiting routine clinical use include heterogeneity in donor microbiota profiles, lack of standardized administration protocols, and uncertainty regarding long-term safety [94]. In addition, practical limitations, such as donor screening burdens and resource demands, continue to restrict its wider application in clinical settings [95].

Given these limitations, further large-scale, long-term clinical trials are required to determine optimal donor selection criteria, dosing strategies, delivery routes, and patient characteristics that predict treatment response. Until such evidence is available, FMT remains a promising but experimental therapeutic strategy for metabolic dysfunction in T2DM.

## 7. Prebiotics, Probiotics, and Postbiotics

Probiotics are defined as live microorganisms that, when administered in adequate amounts, confer health benefits to the host. Frequently used strains include members of the genera *Lactobacillus*, *Bifidobacterium*, *Streptococcus*, and *Saccharomyces*. In patients with T2DM, probiotic supplementation has been shown to improve insulin sensitivity, reduce fasting glucose, and attenuate inflammation. A meta-analysis of randomized controlled trials demonstrated significant reductions in HbA1c and HOMA-IR following probiotic use [96].

In patients with T2DM, probiotics deliver enhanced insulin sensitivity and reduced fasting blood glucose and inflammation. According to a meta-analysis of 12 randomized controlled trials, the application of probiotics substantially decreased HbA1c levels and alleviated insulin resistance [97]. Similarly, a systematic review and meta-analysis of 14 randomized controlled trials (823 participants) found that the use of a probiotic combination of *Lactobacillus acidophilus*, *Lactobacillus casei*, and *Bifidobacterium bifidum* improved HOMA-IR, fasting glucose, insulin concentration, and pancreatic β-cell function, with no evident publication bias [98]. In addition, it has been shown that probiotic administration in a T2DM mouse model inhibited gluconeogenesis [99].

Prebiotics are nondigestible dietary fibers, primarily oligosaccharides, that selectively stimulate the growth of beneficial gut bacteria, such as *Bifidobacterium* and *Akkermansia muciniphila.* They are fermented in the colon to produce SCFAs, which enhance GLP-1 secretion, improve insulin signaling, and strengthen intestinal barrier function.

Among dietary carbohydrates, nondigestible oligosaccharides (NDOs), including some disaccharides, are the primary types of prebiotics. They can be obtained from natural plant and animal sources or produced enzymatically or chemically [100]. These prebiotics are fermented by the gut microbiota to produce SCFAs, including butyrate, which contribute to gut and metabolic health (for mechanisms of SCFAs, see Section 4.1 and Section 5.1) [101,102].

Nondigestible oligosaccharides (NDOs) and other dietary fibers often produce similar physiological effects, including the generation of short-chain fatty acids (SCFAs), suggesting overlapping mechanisms. Prebiotics are distinguished by their ability to selectively stimulate the growth of beneficial bacteria, such as *bifidobacteria* [100]. In individuals with type 2 diabetes, a six-week probiotic or prebiotic intervention increased levels of *Akkermansia muciniphila* and *Faecalibacterium prausnitzii*, accompanied by improved glycemic control, highlighting a potential link between these bacteria and metabolic health [103,104].

Postbiotics are bioactive compounds generated during the fermentation of prebiotics or probiotics, and they include short-chain fatty acids (SCFAs), peptides, exopolysaccharides, enzymes, and structural components of inactivated microbial cells, such as peptidoglycans and lipoteichoic acids. Unlike probiotics, postbiotics do not require the administration of live microorganisms, making them more stable, safer for immunocompromised patients, and easier to standardize for clinical application. Production of postbiotics commonly involves microbial fermentation in proteolytic broths, where maintaining a near-neutral pH optimizes metabolite yield and biological activity. These compounds exert antidiabetic effects through multiple mechanisms: they enhance intestinal barrier integrity by upregulating tight junction proteins, reduce endotoxin (lipopolysaccharide)-induced systemic inflammation, modulate gut-derived hormone secretion, such as GLP-1, and activate AMPK signaling to improve insulin receptor sensitivity. Collectively, postbiotics restore gut–metabolic homeostasis and reduce insulin resistance, positioning them as a promising microbiome-based therapeutic strategy for T2DM. However, most evidence to date arises from experimental and preclinical studies, and well-designed human clinical trials are still needed to validate their long-term metabolic benefits [105,106,107].

## 8. Pharmacological Modulation

### 8.1. Metformin

As outlined in Section 3, dysbiosis contributes to insulin resistance through impaired gut barrier integrity, reduced short-chain fatty acid (SCFA) production, and increased metabolic inflammation. Building on these mechanisms, metformin—long established as the first-line pharmacological treatment for T2DM—exerts part of its therapeutic effect through modulation of the gut microbiota. Metformin increases the abundance of *Akkermansia muciniphila*, *Bifidobacterium*, and SCFA-producing genera such as *Ruminococcus* and *Lactobacillus*. These microbial shifts enhance SCFA availability, strengthen intestinal barrier integrity, and reduce circulating lipopolysaccharide (LPS), thereby lowering metabolic endotoxemia and improving insulin sensitivity.

The microbiome-mediated component of metformin’s action has been demonstrated experimentally, as previously described in Section 3.1, where fecal microbiota transfer from metformin-treated humans improved glucose tolerance in germ-free mice compared to controls, providing causal evidence that metformin-induced changes in microbial composition directly contribute to its metabolic effects [79]. Moreover, metformin has been shown to increase GLP-1 secretion via SCFA and bile acid signaling pathways, further supporting its role in modulating host–microbe interactions.

However, metformin-induced alterations of the gut microbiota have also been linked to gastrointestinal side effects, such as bloating and diarrhea. These adverse effects are thought to arise from increased intestinal fermentation and altered bile acid metabolism. As such, interindividual variability in microbiome composition may contribute to differences in metformin tolerability and treatment response across patients [79].

### 8.2. Glucagon-like Peptide 1 (GLP-1) Agonists

In a systematic review that analyzed 38 studies on the effects of GLP-1 analogs on gut microbiota, GLP-1 agonists were shown to significantly influence the composition, diversity, and richness of the gut microbiota. Liraglutide was shown to support the growth of bacterial genera tied to positive metabolic effects. Both animal and human studies demonstrated positive impacts from the administration of exenatide and exendin-4: in animal models, these agents promoted genera that improve metabolism, while in human studies, they expanded genera associated with improved metabolic function. Dulaglutide caused a significant increase in the abundance of *Bacteroides*, *Akkermansia*, and *Ruminococcus*, which are associated with improved metabolic health. Such result inconsistencies are probably influenced by the variation in the characteristics of study populations and treatment durations. These inconsistencies mainly relate to conflicting findings on whether GLP-1RAs increase or decrease specific bacterial taxa, such as *Akkermansia* and *Bacteroides*, as well as variability in the reported effects on microbial diversity and SCFA production across studies. Thus, further studies are needed to confirm and clarify such findings and their significance for clinical outcomes [108].

### 8.3. Alpha Glucosidase Inhibitors (AGIs)

As part of their therapeutic function, AGIs limit carbohydrate absorption in the small intestine by inhibiting enzymes that digest complex carbohydrates into simple, absorbable forms. Undigested complex carbohydrates can thus be taken up by bacteria in the large intestine and promote the abundance of species, including *Faecalibacterium*, *Bifidobacterium longum*, and *Lactobacillus*, or fermented into SCFAs, as observed in murine models. Increased SCFA production, particularly butyrate and propionate, enhances intestinal barrier integrity and improves insulin signaling through AMPK activation, contributing to improved metabolic control.

Clinical studies have shown that acarbose, the most commonly used AGI, increases microbial diversity and raises the abundance of SCFA-producing bacteria in patients with T2DM, which correlates with reductions in postprandial glucose and inflammatory markers [109].

These increases are attributed to improvements in glycemic control, gut barrier integrity, and metabolic profiles. However, the clinical impact of AGIs on the gut microbiome appears to be dose-dependent, and variability in patient response suggests that baseline microbiota composition may influence treatment outcomes [110,111].

A summary of human clinical trials investigating microbiome-targeted interventions in T2DM is presented in Table 2.

## 9. Future Therapeutic Directions

### 9.1. Next-Generation Probiotics

While interest in probiotics has grown significantly, their actual clinical effectiveness and underlying mechanisms of action are still not well understood [117]. Available market probiotics lack specificity in treating or preventing disease. Probiotic effectiveness can vary widely between individuals as well as across different disease states, which further complicates strain identification for targeted therapeutic use [118]. Probiotics often fail to survive the harsh conditions of the gastrointestinal tract (GIT), limiting their functional impact. These limitations emphasize the need for next-generation probiotics (NGPs) that are specifically designed to target certain health conditions and interact more effectively with the host microbiome [119].

Next-generation probiotics (NGPs) offer the potential for treatment based on an individual’s microbiome composition and the specific targeted disease [120]. NGPs can also be engineered for enhanced survival in the harsh environment of the gastrointestinal tract to ensure greater stability and effectiveness.

Recent advancements in technologies such as high-throughput sequencing and metagenomic sequencing, functional pathway analysis, Genome-Wide Association Studies (GWASs), and transcriptomics have accelerated the identification of novel microbial strains with health-promoting properties, which make them strong candidates for future NGP development [121]. Potential next-generation probiotics include strains such as *Faecalibacterium prausnitzii*, *Akkermansia muciniphila*, and *Bacteroides fragilis*. These probiotic strains have been investigated for their roles in various diseases and health-related conditions [122]. Examples of commonly studied probiotic strains and their primary functions are summarized in Table 3.

### 9.2. Microbiome Editing

Clustered regularly interspaced short palindromic repeats (CRISPR) is a highly effective gene-editing tool that has transformed the field of molecular biology. CRISPR facilitates highly accurate genetic modifications that enhance the performance of probiotics. Using CRISPR, specific genes in probiotic strains can be added, altered, or removed in order to encourage their survival in the gastrointestinal tract or enhance their production of health-promoting compounds [123]. CRISPR/Cas9 was used in a study involving *Akkermansia muciniphila* to promote its production of mucin-degrading enzymes. These enzymes enhance the bacterium’s ability to reinforce the intestinal barrier and lower inflammation, thus improving its effectiveness in managing metabolic conditions such as obesity and diabetes [124]. An overview of therapeutic interventions targeting the gut microbiota and their mechanisms relevant to T2DM is shown in Table 4.

## 10. Current Gaps and Future Research Directions 

Despite the strides made in sequencing technologies and tools, challenges remain at the forefront of further development.

A multitude of confounding factors persist when studying the gut microbiome, particularly diet, geography, and genetics. With these factors come significant challenges to generalizability and identification of a standardized composition. Employing longitudinal or interventional studies may help to clarify correlation versus causation, as is particularly seen in cross-sectional designs, thus allowing a clearer understanding of how composition influences health and disease.

Commonly used in the field of microbial identification, 16S rRNA gene sequencing has been a favored tool, though limited to bacteria and archaea. Large-scale analysis and the lack of a need for culturing allow for rapid results, and the method is a valuable tool for the identification and comparison of species. However, it is still limited to bacteria and archaea and only to certain taxonomic levels [52]. Although shotgun metagenomics may bridge these gaps, its high cost and more technical demands remain barriers.

In an attempt to further analytical methodologies, cost, computational demands, and analytical depth must be considered. Whole-metagenome sequencing, a potential player in the field, also has its own limitations; in mapping the Human Microbiome, a disparity in studied populations severely limits understanding of influencing factors [52].

Though also restricted by disproportionate geographical representation as well as genome gaps, genome-resolved metagenomics could be another approach. Despite challenges in training and technique, incorporating artificial intelligence may be possible in the future [125]; however, new technologies need time to be developed and validated.

## 11. Conclusions

Mediated by effects on insulin resistance, bile acid signaling, inflammation, and glucose and lipid metabolism, the gut microbiome exerts an influence on the development and progression of T2DM. Dysbiosis disrupts immune and metabolic pathways via several mechanisms, including alterations in the production of SCFAs, BCAAs, and bile acids. Medications, diet, genetics, and the environment, as well as probiotics, prebiotics, and certain drugs, all have an impact. Novel therapies, such as FMT, hold some promise but require further validation in future studies. Moving forward, long-term studies tailored toward the microbiome’s role and its potential in supporting metabolic health while addressing the increasing effect of T2DM globally are essential.

## Figures and Tables

**Table 1:** Key microbial metabolites and their effects on host metabolism.

Metabolite	Host Target	Physiological Effect
SCFAs (acetate, butyrate, propionate)	FFAR2/FFAR3, colonocytes	Improves insulin sensitivity, enhances barrier integrity
BCAAs (leucine, isoleucine, valine)	Liver, skeletal muscle	Impairs insulin signaling, activates mTOR
Secondary bile acids	FXR, TGR5	Modulates GLP-1, glucose, and lipid homeostasis
LPS	TLR4	Triggers inflammation and insulin resistance

**Table 2:** Clinical trials and human studies targeting the gut microbiota in T2DM.

Intervention	Study Design/Population	Duration	Primary Outcomes	Microbiome Effect	References
Inulin-type fructans (FOS/inulin)	RCT in women with T2DM	8 weeks	↓ Fasting glucose, ↓ HbA1C, ↓ Endotoxemia (LPS)	↑ *Bifidobacterium*, ↑ SCFAs	[65]
Probiotics (Lactobacillus/Bifidobacterium strains)	Meta-analysis of RCTs in T2DM (12 trials)	—	↓ HbA1c, ↓ HOMA-IR; improved fasting glucose	↑ gut diversity, ↓ inflammatory species	[112]
Synbiotics	RCTs in T2DM	8–12 weeks	↓ HOMA-IR, ↓ inflammation	Synergistic increase in SCFA producers	[113]
FMT (lean donor → recipients with metabolic syndrome)	Double-blind, controlled	6 weeks	↑ Peripheral insulin sensitivity; ↑ butyrate producers (Roseburia)	↑ *Roseburia*, ↑ butyrate-producing taxa	[67]
Metformin (microbiome-linked effects)	Human cohorts/intervention	—	↑ *Akkermansia muciniphila*, ↑ SCFA-producing taxa; improved glycemic control; GI intolerance varies by microbiome profile	↑ *Akkermansia*, ↑ SCFAs	[79]
GLP-1 receptor agonists	Systematic review of animal + human studies	—	↑ Microbial diversity; shifts in *Akkermansia*, *Bacteroides*, *Ruminococcus*	↑ diversity; shifts in *Akkermansia* and *Bacteroides*	[108]
Dietary fiber/Mediterranean diet	Cohorts and interventions	8–24 weeks	↑ SCFAs; ↓ HbA1c; improved TG/HDL	↑ SCFAs, ↑ microbial diversity	[114,115,116]

**Table 3:** Common probiotic strains and their associated functions.

Probiotic Strain	Health Benefit
*Lactobacillus acidophilus*	Enhances the gut barrier, suppresses pathogens
*Bifidobacterium longum*	Anti-inflammatory, reduces gut permeability
*Streptococcus thermophilus*	Aids lactose digestion, modulates immunity
*Saccharomyces boulardii*	Prevents diarrhea, supports microbiota recovery
*Escherichia coli Nissle 1917*	Competes with pathogens, maintains balance

**Table 4:** Therapeutic interventions and their effects on the gut microbiota in T2DM.

Intervention	Mechanism of Action	Microbiota Impact
Prebiotics	Stimulate growth of beneficial bacteria	Increase SCFA producers
Probiotics	Introduce live beneficial microbes	Restore microbial diversity
FMT	Transplant fecal microbiota from healthy donor	Re-establish eubiosis
Dietary fiber	Substrate for microbial fermentation	Boosts butyrate production
Metformin	Modifies gut microbiota composition	Enriches Akkermansia and SCFA-producing bacteria

## Data Availability

No new data were created or analyzed in this study. Data sharing is not applicable to this article.
